# Down-regulation of Sp1 suppresses cell proliferation, clonogenicity and the expressions of stem cell markers in nasopharyngeal carcinoma

**DOI:** 10.1186/s12967-014-0222-1

**Published:** 2014-08-07

**Authors:** Jing-Ping Zhang, Hua Zhang, Hong-Bo Wang, Yan-Xian Li, Gui-Hong Liu, Shan Xing, Man-Zhi Li, Mu-Sheng Zeng

**Affiliations:** 1 State Key Laboratory of Oncology in South China, Sun Yat-sen University Cancer Center, 651 Dongfeng Road East, Guangzhou 510060, China; 2 Department of Experimental Research, Sun Yat-sen University Cancer Center, Guangzhou, China; 3 Department of Oncology, the Second Affiliated Hospital of Guangzhou medical college, Guangzhou, China

**Keywords:** Nasopharyngeal carcinoma, Sp1, Cell cycle, Cell proliferation, Clonogenicity, Stem cell

## Abstract

**Background:**

Transcription factor Sp1 is multifaceted, with the ability to function as an oncogene or a tumor suppressor, depending on the cellular context. We previously reported that Sp1 is required for the transcriptional activation of the key oncogenes in nasopharyngeal carcinoma (NPC), including B-lymphoma mouse Moloney leukemia virus insertion region 1 (Bmi1) and centromere protein H (CENPH), but the role of Sp1 and its underlying mechanisms in NPC remained largely unexplored. The objective of this study was to investigate the cellular function of Sp1 and to verify the clinical significance of Sp1 as a potential therapeutic target in NPC.

**Methods:**

The levels of Sp1 in the normal primary nasopharyngeal epithelial cells (NPECs) and NPC cell lines were analyzed by Quantitative Real-time RT-PCR (qRT-PCR) and Western blot. The location and expression of Sp1 in the NPC tissues were detected by immunohistochemistry staining (IHC). The effect of Sp1 knockdown on the cell proliferation, clonogenicity, anchorage-independent growth and the stem-cell like phenotype in NPC cells were evaluated by MTT, flow cytometry, clonogenicity analysis and sphere formation assay.

**Results:**

The mRNA and protein levels of Sp1 were elevated in NPC cell lines than in the normal primary NPECs. Higher expression of Sp1 was found in NPC tissues with advanced clinical stage (*P* = 0.00036). Either inhibition of Sp1 activity by mithramycin A, the FDA-approved chemotherapeutic anticancer drug or Sp1 silencing by two distinct siRNA against Sp1 suppressed the growth of NPC cells. Mechanism analysis revealed that Sp1 silencing may suppress cell proliferation, clonogenicity, anchorage-independent growth and the stem-cell like phenotype through inducing the expression of p27 and p21, and impairing the expressions of the critical stem cell transcription factors (SCTFs), including Bmi1, c-Myc and KLF4 in NPC cells.

**Conclusions:**

Sp1 was enriched in advanced NPC tissues and silencing of Sp1 significantly inhibited cell proliferation, clonogenicity, anchorage-independent growth and the stem-cell like phenotype of NPC cells, suggesting Sp1 may serve as an appealing drug target for NPC.

## Background

Nasopharyngeal carcinoma (NPC) is one of the leading malignancies in southern China and Southeast Asia [1],[2]. The annual incidence rate reaches 30 cases per 100,000 people in the endemic regions, which is about 25-fold higher than in the rest of the world [1]–[3]. NPC shows familial clustering, resulting predominantly from inherited susceptibility [4]. Elevated levels of serum Epstein-Barr virus (EBV)-related antibodies, circulating and intratumoral EBV DNA were consistently detected in NPC individuals, demonstrating that EBV infection is a strong risk factor for NPC [5]–[7]. In addition, smoking and consumption of salt-preserved foods have also been reported to be the moderate risk factors for NPC [8],[9]. Therefore, NPC is a malignancy with a complex etiology involving both genetic and environmental factors [1]–[3]. Although radiotherapy or chemo-radiotherapy has been acknowledged as standard treatment options, these treatments induce a considerable incidence of acute mucosal and hematologic toxicity [10]–[12]. Thus, it is urgent to identify the potential therapeutic agents for NPC.

Sp1 was identified as a transcription factor by binding to the promoters of its target genes and has been postulated to play important roles in the regulation of multiple critical biological functions, including cell apoptosis [13] and growth [14], cycle progression [15], purine/pyrimidine synthesis [16], metabolism [17], angiogenesis [18],[19] and metastasis [20],[21]. Growing evidence demonstrated Sp1 is abnormally expressed in a variety of epithelial tumors originating from different tissues, including breast [22], thyroid [23], pancreas [24], stomach [25] and lung [26]. Deregulation of Sp1 may promote tumor development through regulating transcription of genes, such as c-Myc [27], cyclin D1 [28], VEGF [29] and MMP9 [30]. In contrast, overexpression of Sp1 in highly invasive lung adenocarcinoma cells increases expression of E-cadherin and inhibits metastasis [20]. Moreover, Sp1 induces apoptosis through cooperating with p53 [31]. Therefore, Sp1 may serve as an oncogene or a tumor suppressor in tumor development and deserved to be studied in depth.

We previously identified Sp1 as an important transcription factor for oncogenes in NPC, including B-lymphoma mouse Moloney leukemia virus insertion region 1 (Bmi1) and centromere protein H (CENPH), but the role of Sp1 in the development of NPC remains obscure [32],[33]. In the present study, we found the level of Sp1 was elevated in advanced NPC tissues and silencing of Sp1 significantly inhibited cell proliferation, clonogenicity, anchorage-independent growth and the stem-cell like phenotype of NPC cells, suggesting Sp1 as a potential therapeutic target for NPC.

## Materials and methods

### Reagents

The reagents used were as follows: rabbit polyclonal antibodies against Sp1 (#07-645, Millipore, Temecula, CA, USA), p27 (#60-645, Millipore), CDK4 (ab137818, Abcam, Cambridge, MA, USA); mouse monoclonal antibodies (mAb) against p21 (sc-6246, Santa Cruz, CA, USA), α-tublin (T6199, Sigma-Aldrich, St Louis, MO, USA), GAPDH (sc-32233, Santa Cruz). All other reagents were obtained from Sigma-Aldrich, unless otherwise indicated.

### Patients and tissue specimens

The paraffin-embedded NPC specimens (n = 76) were obtained from Cancer Center of Sun Yat-sen University in Guangzhou, People’s Republic of China. Written informed consent was obtained from each patient, and the study was approved by the Institute Research Ethics Committee at the Cancer Center. The clinical characteristics of the NPC patients were described in Table 1. All cases were histologically confirmed.

**Table 1:** Clinical characteristics of 76 NPC patients

**Characteristics**	**No. (%)**
**Gender**	
Female	21 (27.6%)
Male	55 (72.4%)
**Age (y)**	
≤ 45	34 (44.7%)
> 45	42 (55.3%)
**Histological classification**	
NKUC	74 (97.4%)
NKDC	2 (2.6%)
**Clinical stage**	
I	1 (1.3%)
II	23 (30.3%)
III	27 (35.5%)
IV	25 (32.9%)
**T classification**	
T1	10 (13.2%)
T2	25 (32.9%)
T3	18 (23.7%)
T4	23 (30.3%)
**N classification**	
N0	17 (22.4%)
N1	30 (39.5%)
N2	26 (34.2%)
N3	3 (3.9%)
**Metastases**	
No	74 (97.4%)
Yes	2 (2.6%)
**Expression of Sp1**	
Low	33 (43.4%)
High	43 (56.6%)

*NPC* nasopharyngeal carcinoma.

*NKUC* non-keratinizing undifferentiated carcinoma.

*NKDC* non-keratinizing differentiated carcinoma.

### Cell culture

Normal primary NPECs (NPECw and NPEC01) were established as described previously [34], and cultured in keratinocyte/serum-free medium (Invitrogen, Carlsbad, CA, USA). The NPC cell lines (CNE2, HNE1, HONE1, C666-1 and HK1) were cultured in RPMI 1640 (Invitrogen, Carlsbad, CA) supplemented with 10% fetal bovine serum (FBS; Hyclone, Logan, UT), and in a humidified 5% CO2 incubator at 37°C.

### SiNRA transfection

The siRNA oligoribonucleotides were purchased from Dharmacon (Rockford, USA) and Ribobio (Ribobio, Guangzhou, China). The siRNAs targeting the mRNA of human Sp1 [GenBank: NM_138473.2] was denoted as siSp1-1# (l-026959-00-0003) and siSp1-2# (siG0898155658). The negative control (NC) RNA duplex for the siRNA was nonhomologous to any human genome sequences, and was indicated as siNC (D-001220-01-20). CNE2 and HNE1 cells (1 × 10^5^) were seeded on 6-well plates for 16 h, followed by transfection with 50 nM of the RNA duplex and 5 μL of Lipofectamine RNAiMAX (Invitrogen, Grand Island, NY, USA), according to the manufacturer’s instructions. After 72 h, cells were washed with PBS and harvested for further experiments.

### Quantitative real-time RT-PCR

Total RNA from NPC cell lines or tissue specimens was extracted using the TRIzol reagent (Invitrogen, Grand Island, NY, USA) according to the manufacturer’s instructions. Complementary DNA (cDNA) was synthesized from 1 μg of the total RNA using a reverse transcriptase kit (Invitrogen). The mRNA level was determined by quantitative Real-time RT-PCR (qRT-PCR) using the Power SYBR Green qPCR SuperMix-UDG (Invitrogen, Grand Island, NY, USA) and was analyzed on an ABIPrism-7500 Sequence Detector System (ABI, applied biosystems, USA). Glyceraldehyde-3-phosphate dehydrogenase (GAPDH) or β-actin was used as an internal control. The primers are listed in Table 2. Relative expression of the indicated gene was normalized to GAPDH or β-actin expression, which yielded a 2^-△△Ct^ value. All reactions were run in triplicate.

**Table 2:** Sequences of primers

**Name**	**Sense primer (5′ - 3′)**	**Antisense primer (5′ - 3′)**
*SP1*	TCCAGACCATTAACCTCAGTGC	TGTATTCCATCACCACCAGCC
*p21*	5′-CCTGTCACTGTCTTGTACCCT	GCGTTTGGAGTGGTAGAAATCT
*p27*	CTGCAACCGACGATTCTTCTACT	GGGCGTCTGCTCCACAGA
*BMI1*	TGGCTCGCATTCATTTTCTG	TGTGGCATCAATGAAGTACCCT
*MYC*	GGAGGCTATTCTGCCCATTTG	CGAGGTCATAGTTCCTGTTGGTG
*KLF4*	CACAAAGAGTTCCCATCTCAAGGC	CGGTAGTGCCTGGTCAGTTCATC
*ABCG2*	CATGTACTGGCGAAGAATATTTGGT	CACGTGATTCTTCCACAAGCC
*OCT4*	GTGGAGAGCAACTCCGATG	TGCTCCAGCTTCTCCTTCTC
*GAPDH*	CTCCTCCTGTTCGACAGTCAGC	CCCAATACGACCAAATCCGTT
*Actin*	CGCGAGAAGATGACCCAGAT	GGGCATACCCCTCGTAGATG
*SOX2*	CGAGTGGAAACTTTTGTCGGA	TGTGCAGCGCTCGCAG

### Western blot

Western blot was performed as previously described [32]. Briefly, cells were harvested and lysed in RIPA buffer containing protein inhibitor cocktail (F. Hoffmann-La Roche Ltd, Basel, Switzerland). The protein concentration was determined by the BCA method (Pierce, Rockford, IL). The protein (10 μg) was electrophoretically separated in 9 or 12% SDS polyacrylamide gels, and transferred to polyvinylidene difluoride membranes (Millipore). After probing with the indicated antibodies, the signals were detected using enhanced chemiluminescence (ECL) (Amersham Pharmacia Biotech, Piscataway, NJ). The same membranes were stripped and reprobed with mouse monoclonal antibodies against GAPDH or α-tubulin to confirm equal loading of the samples.

### Immunohistochemical staining (IHC)

Immunohistochemical staining (IHC) was performed as previously described [34]. Briefly, paraffin-embedded samples were cut into 4 μm sections and placed on polylysine-coated slides. Paraffin sections were baked for 2 h at 58°C, de-paraffinized in xylene, rehydrated through graded ethanol, quenched for endogenous peroxidase activity in 0.3% hydrogen peroxide for 10 min, and processed for antigen retrieval by high pressure cooking in citrate antigen retrieval solution (pH = 6.0) for about 5 min. Sections were incubated at 4°C overnight with rabbit polyclonal antibodies against Sp1 (Millipore, 1:3200) in a moist chamber. Immunostaining was performed using the Zymed Histostain TM­Plus Kits (Zymed, South San Francisco, CA, USA), which resulted in a brown-colored precipitate at the antigen site. Subsequently, sections were counterstained with hematoxylin (Zymed Laboratories, South San Francisco, CA) and mounted in a non-aqueous mounting medium. All runs included a no primary antibody control. The immunohistochemically stained tissue sections were scored separately by two pathologists blinded to the clinical parameters. The staining intensity was scored as 0 (negative), 1 (weak), 2 (medium) or 3 (strong). Extent of staining was scored as 0 (0-10%), 1 (10-25%), 2 (26-50%) and 3 (51%-100%) according to the percentages of the positive staining areas in relation to the whole carcinoma area. The product of the intensity and extent score was used as the final staining score (0–9) for Sp1. Tumors having a final staining score of < 4.5 were considered to be low and those with score of ≥ 4.5 were considered to be high.

### MTT assay

Forty-eight hours after transfection with siSp1-1#, siSp1-2# or siNC, CNE2 and HNE1 cells were plated in triplicate at 2000 cells per well in 96-well plates and maintained in RPMI containing 10% FBS. At the indicated time points, 10 μL of 5 mg/mL MTT (3- (4, 5-dimethylthiazol-a-yl)-2, 5-diphenyl tetrazolium bromide) was added to each well, and cells were incubated for 4 h at 37°C. The media was then discarded, and 200 μl of DMSO was added to each well for 20 min at RT to dissolve the precipitates. Absorbance was measured using the Spectramax M5 (Molecular Devices, CA, USA) at a wavelength of 570 nm.

### Colony formation assay

Forty-eight hours after transfection with siSp1-1#, siSp1-2# or siNC, CNE2 and HNE1 cells were plated in triplicate at 200 cells per well in six-well plates and maintained in RPMI containing 10% FBS for 10 days. Colonies were fixed with methanol and stained with 0.1% crystal violet in 20% methanol, and then were photographed and counted. All visible colonies were quantified.

### Anchorage-independent growth

Forty-eight hours after transfection with the indicated RNA duplex, CNE2 and HNE1 cells (1 × 10^4^) that were suspended in 0.33% top agar (in RPMI with 10% FBS) were plated onto 0.55% base agar (in RPMI with 5% FBS) in six-well plates for 14 days. The colonies with the diameter bigger than 10 μm were counted.

### Sphere formation assay

Forty-eight hours after transfection with the indicated RNA duplex, CNE2 cell (5 × 10^2^) that were suspended in tumor sphere medium containing serum-free DMEM/F-12, N_2_ supplement, 10 ng/mL human recombinant bFGF and 10 ng/mL EGF were plated in six-well plates (ultra low attachment surface) for 7 days. Colonies were photographed at 200× magnification and were counted at 40× magnification.

### Statistical analysis

All data analysis was conducted with GraphPad Prism version 4.0 (GraphPad Software, San Diego, CA, USA) and SPSS 16.0 (SPSS, Chicago, IL, USA). The results were representative of at least three independent experiments. Data were presented as the mean ± standard error of the mean (SEM) obtained with triplicate samples. Analysis of the differences between groups was determined with the two-tailed Mann–Whitney test. A *P*-value of 0.05 was used as the criterion of statistical significance.

## Results

### High level of Sp1 correlates with tumor progression in nasopharyngeal carcinoma

**Figure 1:**
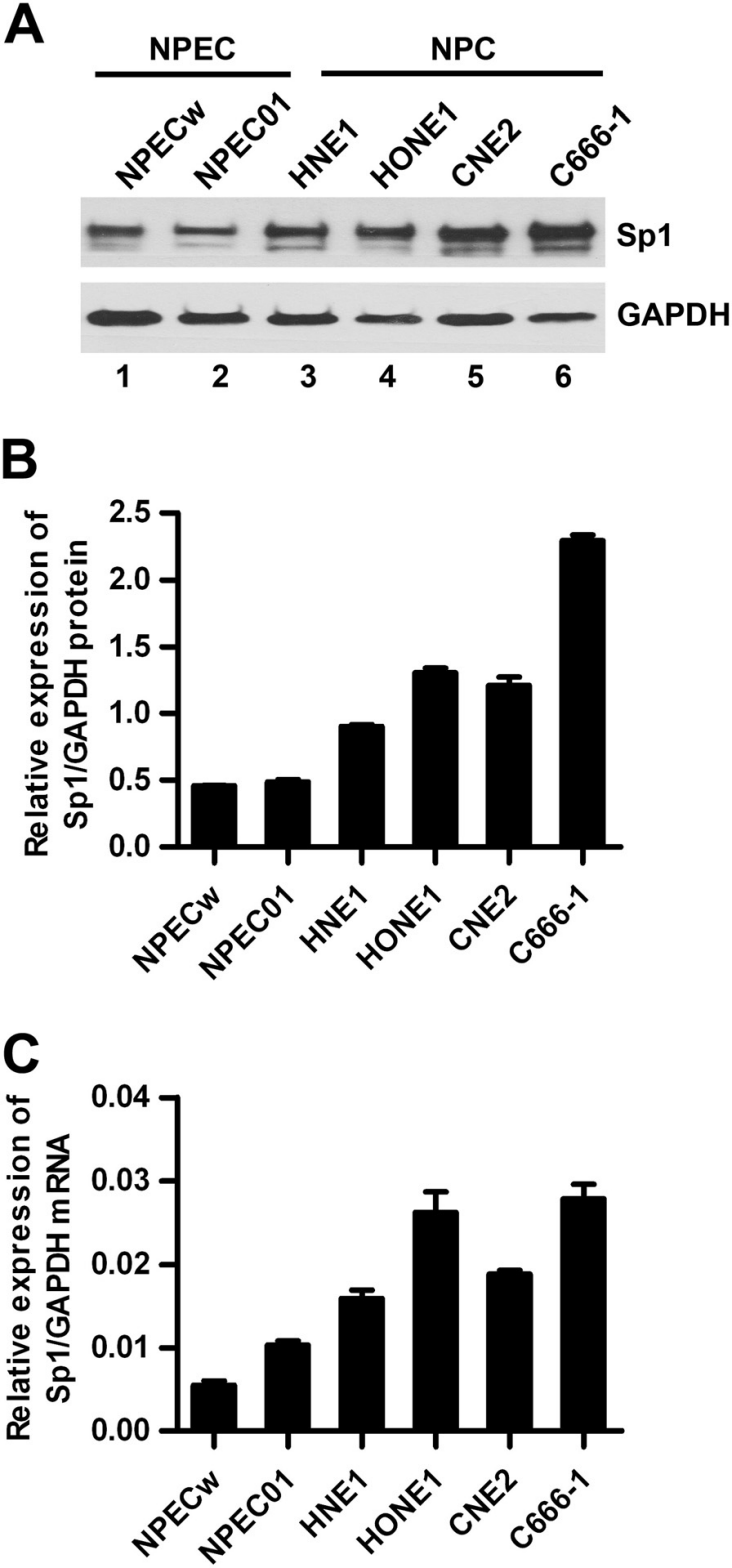
**Expression of Sp1 is higher in NPC cell lines than the normal primary NPECs.** Sp1 protein and mRNA levels were analyzed by Western blot **(A and B)** and qRT-PCR **(C)** in the normal primary NPECs (NPECw and NPEC01), and NPC cell lines (HNE1, HONE1, CNE2 and C666-1). These results were reproducible in three independent experiments and the representative immunoblots were shown in A. Both the protein and mRNA levels of Sp1 were normalized to GAPDH.

**Figure 2:**
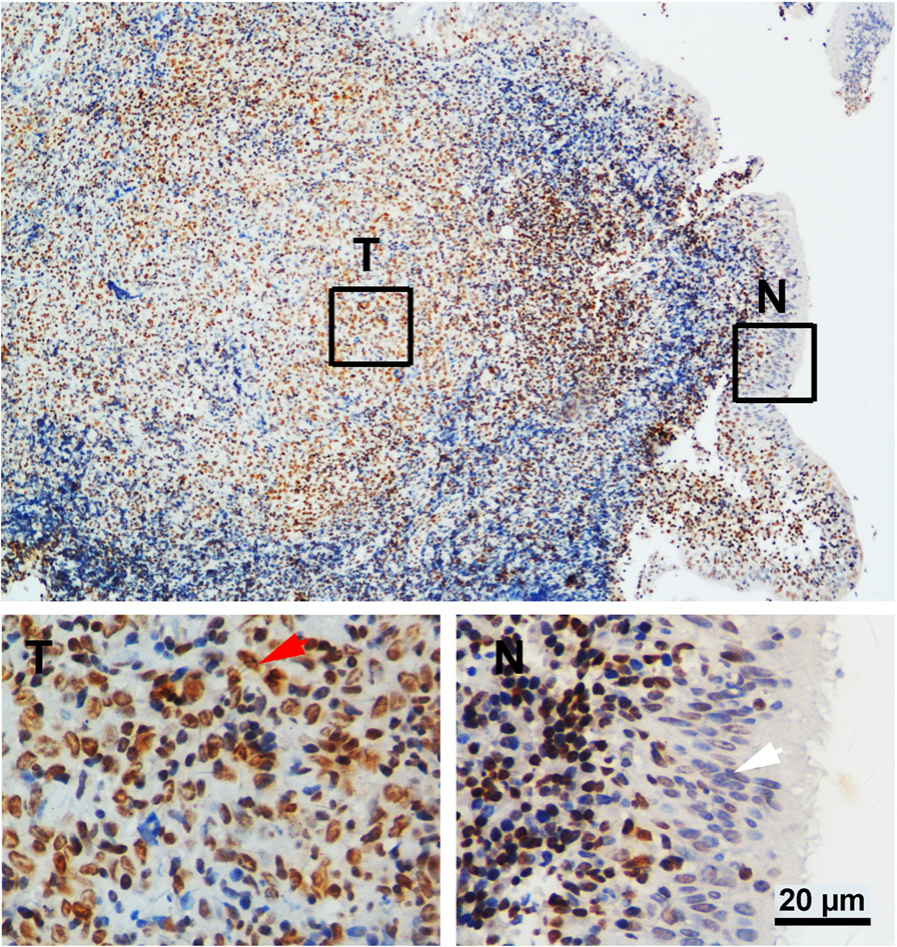
**Expression of Sp1** ***in situ*** **tumors of NPC patients.** Sp1-positive cells were stained brown and present in the nuclei of tumor cells and some lymphocytes. Strong positive staining for Sp1 in NPC cells of the tumor region (T) was marked as red arrow (lower left panel), while negative or weak staining for Sp1 in NPEC of the adjacent non-tumor region (N) was indicated as white arrow (lower right panel). Representative images with low (100×, upper panels) and high (400×, lower panels) magnification were shown. Scale bar = 20 μm.

To explore the significance of Sp1 in the development of nasopharyngeal carcinoma, the mRNA and protein levels of Sp1 were first examined in the normal primary nasopharyngeal epithelial cells (NPECs) and NPC cell lines. Both qRT-PCR and Western blot showed that the level of Sp1 in the four NPC cell lines (HNE1, HONE1, CNE2 and C666-1) was obviously higher than the normal primary NPECs (NPECw and NPEC01) (Figure 1). The expression and subcellular localization of Sp1 in 76 NPC tissues were then determined by immunohistochemistry. As shown in Figure 2, Sp1 was expressed predominantly in the nuclei of NPC cells of the tumor regions (T), but demonstrated weak signal in the NPEC of the non-tumor regions (N). In addition, Sp1 was present in some infiltrating lymphocytes of both the non-tumor and tumor regions of NPC patients. To evaluate the clinical significance of Sp1 in nasopharyngeal carcinoma, the correlation between Sp1 level and the clinical variations was analyzed using *χ*^2^ test. As shown in Table 3, the expression of Sp1 was significantly associated with the clinical stage (*P* = 0.00036), T (*P* = 0.002) and N (*P* = 0.034) classification. There was no significant correlation between the expression level of Sp1 and age, gender, M classification as well as histologic classification in NPC patients. These results suggested that the increase in intratumoral Sp1-positive NPC cells was associated with NPC progression.

**Table 3:** Correlation between the clinical characteristics and Sp1 expression in NPC patients

**Characteristics**	**Sp1**		***P***
	**Low**	**High**	
**Age (y)**			
≤ 45	18 (54.5%)	16 (37.2%)	0.165
> 45	15 (45.5%)	27 (62.8%)	
**Gender**			
Male	24 (72.7%)	31 (72.1%)	1.01
Female	9 (27.3%)	12 (27.9%)	
**Clinical stage**			
I-II	18 (54.5%)	6 (14%)	0.00036
III-IV	15 (45.5%)	37 (86.0%)	
**T classification**			
T1-T2	22 (66.7%)	13 (30.4%)	0.002
T3-T4	11 (33.3%)	30 (69.6%)	
**N classification**			
N0-N1	25 (75.8%)	22 (51.2%)	0.034
N2-3	8 (24.2%)	21 (48.8%)	
**Metastases**			
No	33 (100%)	41 (95.3%)	0.502
Yes	0	2 (4.7%)	
**Histological classification**			
NKUC	31 (93.9%)	43 (100%)	0.185
NKDC	2 (6.1%)	0	
Fisher’s exact test			

### Knockdown of Sp1 suppresses the proliferation of NPC cells

**Figure 3:**
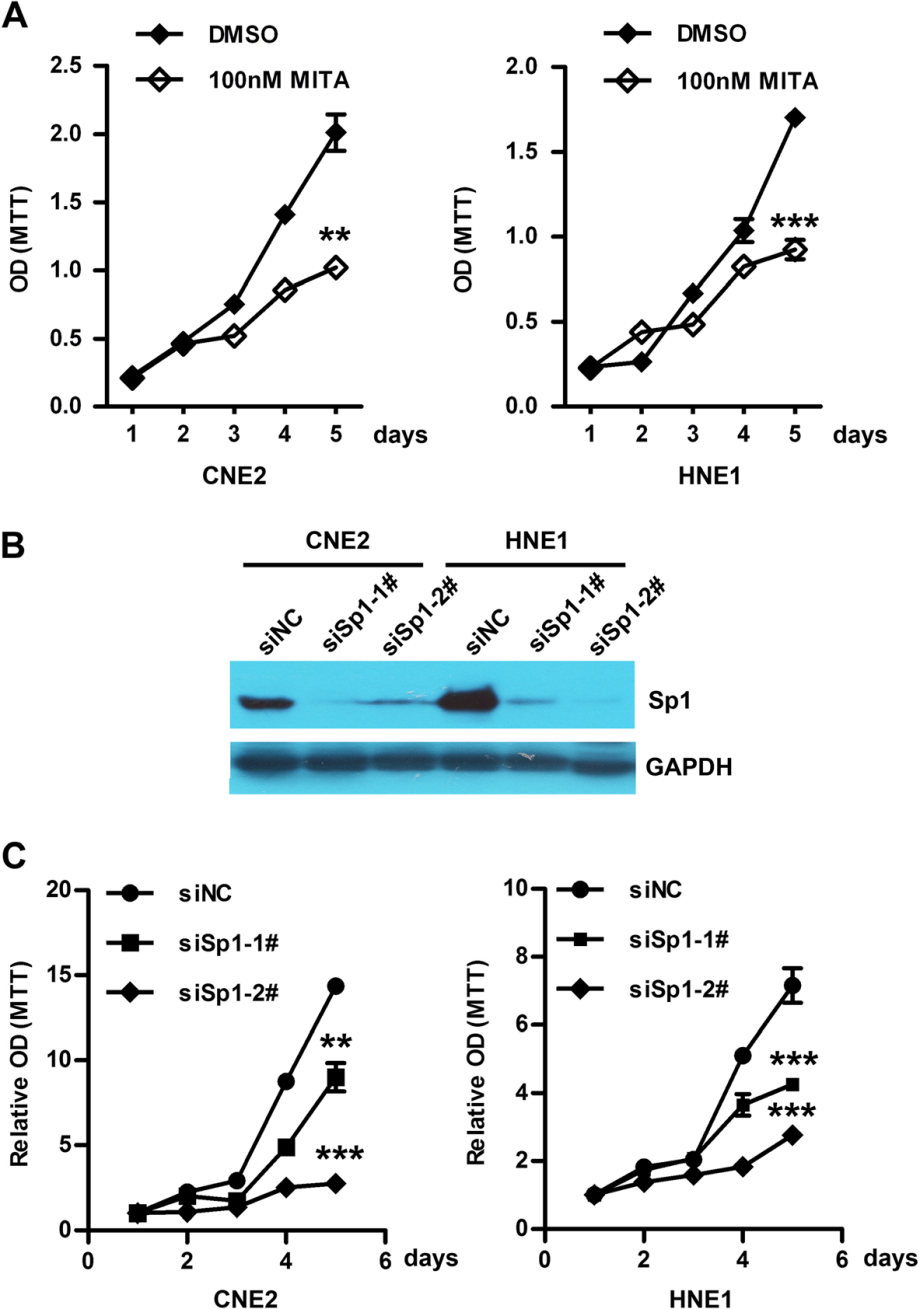
**Both impaired Sp1 activity and expression suppressed the cell viability of NPC cells. (A)** Inhibition of Sp1 activity by MITA treatment decreased the cell viability of CNE2 and HNE1 cells. Twenty-four hours after seeding in 96-well plates, CNE2 and HNE1 cells were treated with 100 nM MITA. The cell viability was determined by MTT assay at the indicated times. ** *P* < 0.01 and *** *P* < 0.001. **(B)** Sp1 was completely abolished in CNE2 and HNE1 cells. After transfection with siSp1-1#, siSp1-2# or siNC for 72 h, cells were subjected to Western blot analysis for Sp1. GAPDH was used as an internal control. **(C)** Silencing of Sp1 impaired the cell viability of CNE2 and HNE1 cells. After transfection with siSp1-1#, siSp1-2# or siNC for 48 h, CNE2 and HNE1 cells were seeded into 96-well plates (2000/well) and incubated for the indicated times. The cell viability was determined by MTT assay at the indicated times. *** *P* < 0.001.

To investigate the potential roles of Sp1 in tumorigenesis, the effect of Sp1 on the cell growth was first evaluated. CNE2 and HNE1 cells were cultured in the presence of 100 nM mithramycin A (MITA), a FDA-approved chemotherapeutic anticancer drug that inhibits transcriptional activity of Sp1 by competitively binding to the Sp1-binding sites. The cell viability was determined by MTT on the indicated times. Compared with DMSO-treated cells, the cell viability was significantly suppressed in MITA-treated CNE2 and HNE1 cells (Figure 3A).

Next, we assessed the effect of Sp1 knockdown on the cell growth of NPC cells. HNE1 and CNE2 cells were transfected with the two distinct siRNA duplexes against Sp1, and then subjected to Western blot. The level of Sp1 was nearly abolished in siSp1 transfectants (Figure 3B). Compared to the control, siSp1 transfectants displayed significantly lower proliferation, suggesting the growth-promoting role of Sp1 in NPC cells (Figure 3C).

### Knockdown of Sp1 suppressed the G1/S phase transition and increased the expressions of p21 and p27

**Figure 4:**
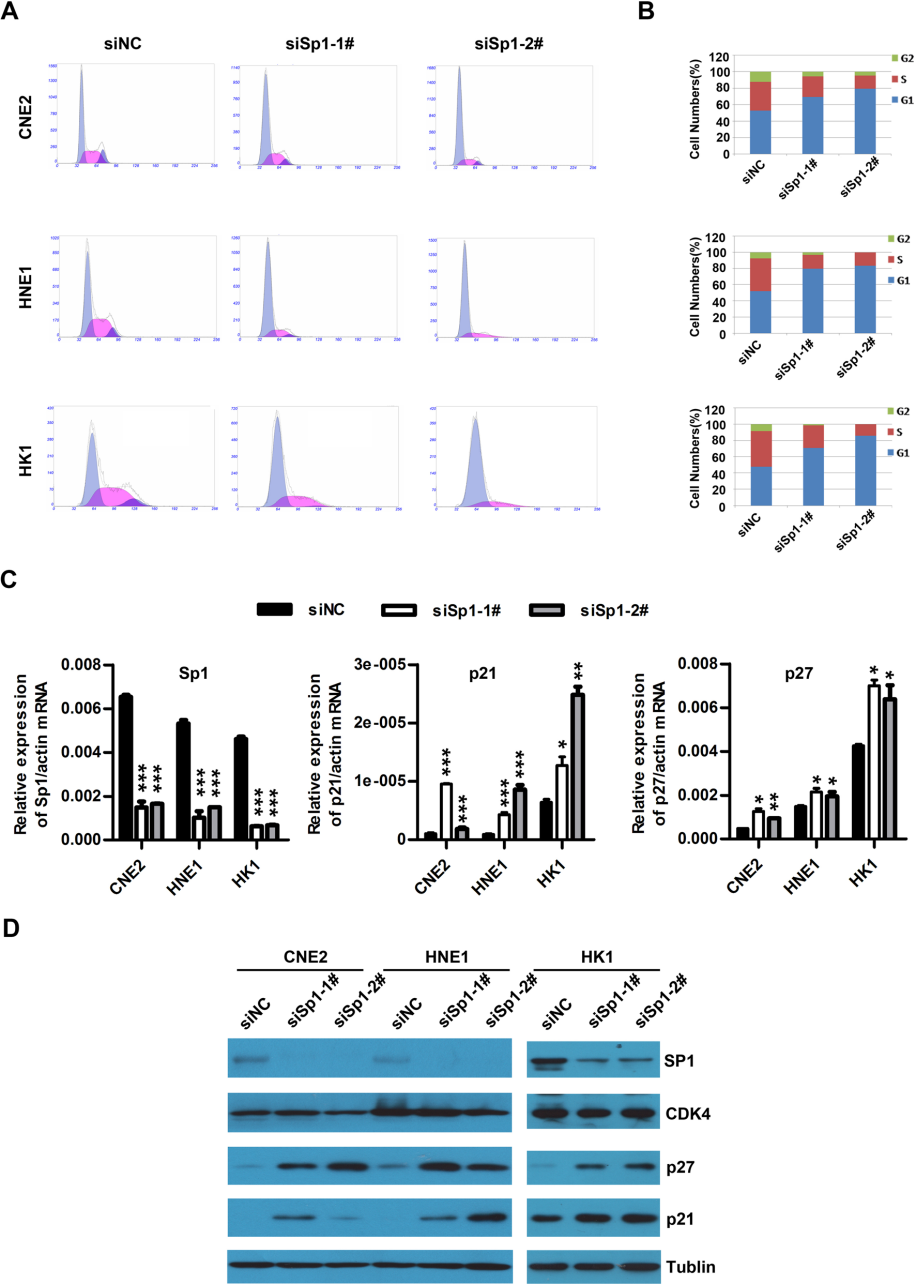
**Knockdown of Sp1 induces G1 phase arrest and up-regulates the expressions of p21 and p27. (A and B)** Knockdown of Sp1 caused accumulation of G1 population. The representative images are shown in A and the percentage of cell distribution in G1/G0, S and G2/M is illustrated in B. CNE2, HNE1 and HK1 cells were plated in 6-well culture plates and transfected with siSp1-1#, siSp1-2# or siNC for 72 h. Cells were harvested, fixed, stained with propidium iodide and then analyzed for DNA content by flow cytometry. * *P* < 0.05, ** *P* < 0.01, *** *P* < 0.001. **(C and D)** Knockdown of Sp1 enhanced the expressions of p27 and p21. CNE2, HNE1 and HK1 cells transfected with siSp1-1#, siSp1-2# or siNC for 72 h were analyzed for the levels of cell cycle related genes by qRT-PCR **(C)** and Western blot **(D)**. For **(C)**, the mRNA level of the indicated gene was normalized to β-actin. * *P* < 0.05, ** *P* < 0.01, *** *P* < 0.001. For **(D)**, α-tubulin served as an internal control.

To assess whether cell cycle progression was involved in Sp1-promoted cell growth, CNE2, HNE1 and HK1 cells were transfected with the indicated RNA duplex for 48 h, followed by flow cytometry analysis for the cell cycle distribution of initially asynchronous cells. Knockdown of Sp1 showed a marked induction of G1 arrest in CNE2, HNE1 and HK1 cells, whereas had no effect on apoptosis as evaluated by sub-G1 peak (Figure 4A and B). The G1/S-phase transition has been characterized as a balance of cyclins, CDKs and Cip1 proteins. Cyclins promote S phase, whereas p27 and p21 maintain cells in G1 arrest. CDK4, p27 and p21 have been reported to regulate the G1/S-phase transition and are potentially regulated by Sp1 [35]–[37]. To study whether Sp1 promotes cell cycle through the expression regulation of these molecules in nasopharyngeal carcinoma, CNE2, HNE1 and HK1 cells were transfected with siSp1 and siNC for 48 h, followed by analysis for the levels of Sp1, CDK4, p27 and p21. Either qRT-PCR or Western blot demonstrated that Sp1 knockdown obviously up-regulated the levels of p27 and p21, but had no effect on the expression levels of CDK4 (Figure 4C and D), indicating Sp1 knockdown blocked the G1/S transition through induction of p27 and p21 in NPC cells.

### Knockdown of Sp1 suppressed clonogenicity and anchorage-independent growth

**Figure 5:**
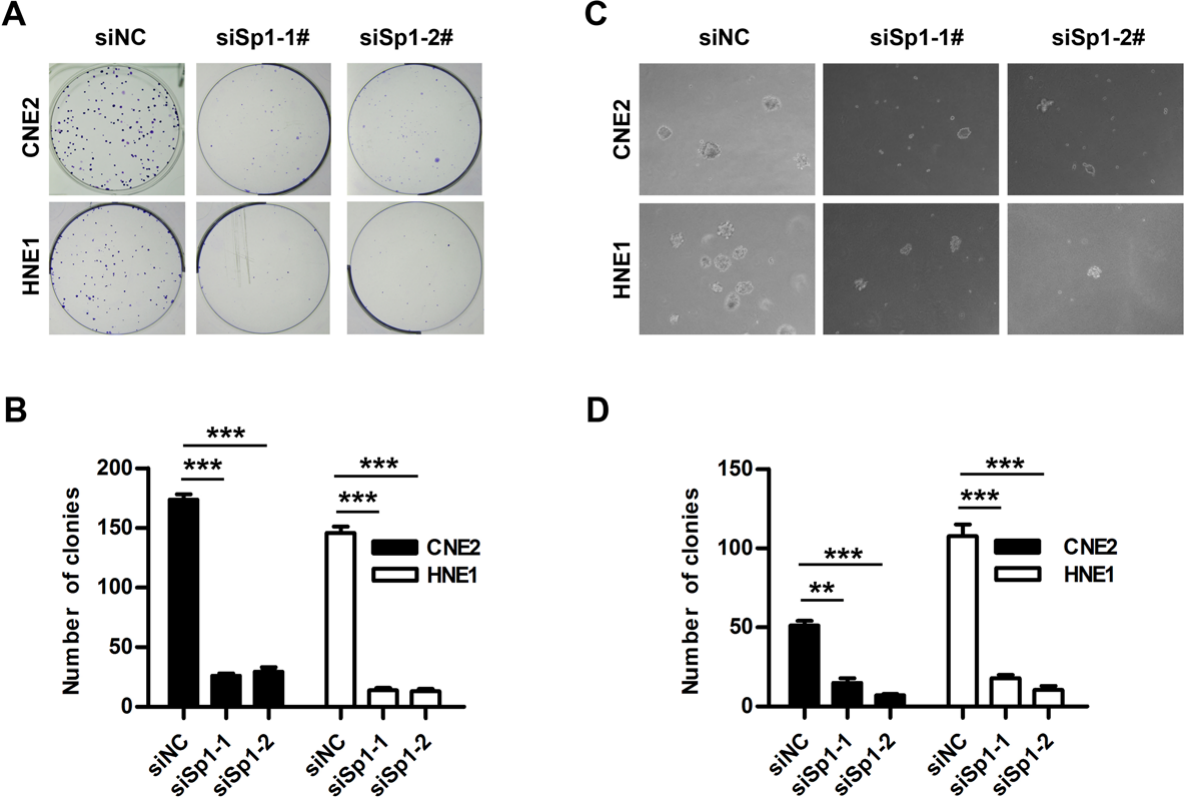
**Knockdown of Sp1 suppressed the clonogenicity and anchorage-independent growth of NPC cells.** CNE2 and HNE1 cells transfected with either siSp1-1#, siSp1-2# or siNC were grow at low density for 10 days **(A and B)** or were plated onto 0.55% base agar (in RPMI with 5% FBS) in six-well plates for 14 days. For **(A and B)** Colonies were fixed with methanol and stained with 0.1% crystal violet in 20% methanol, and then were photographed and counted. All visible colonies were quantified. For **(C and D)**, the colonies with the diameter bigger than 100 μm were counted. ** *P* < 0.01, *** *P* < 0.001.

We then analyzed the effect of Sp1 on the clonogenicity and anchorage-independent growth of NPC cells. CNE2 and HNE1 cells were transfected with the indicated RNA duplex, and then allowed to grow at very low density or grow in soft agar. The siSp1-transfectants displayed obviously fewer and smaller colonies than the siNC-transfectants, as shown by a significant reduction in both the size and the number of colonies (Figure 5A and B). In addition, knockdown of Sp1 significantly suppressed the capacity of NPC cells to grow in soft agar (Figure 5C and D). These results indicated that Sp1 silencing exerted a suppressive role on the colony formation and anchorage-independent growth of NPC cells.

### Sp1 silencing inhibited sphere formation and impaired the expressions of stem cell markers in NPC cells

**Figure 6:**
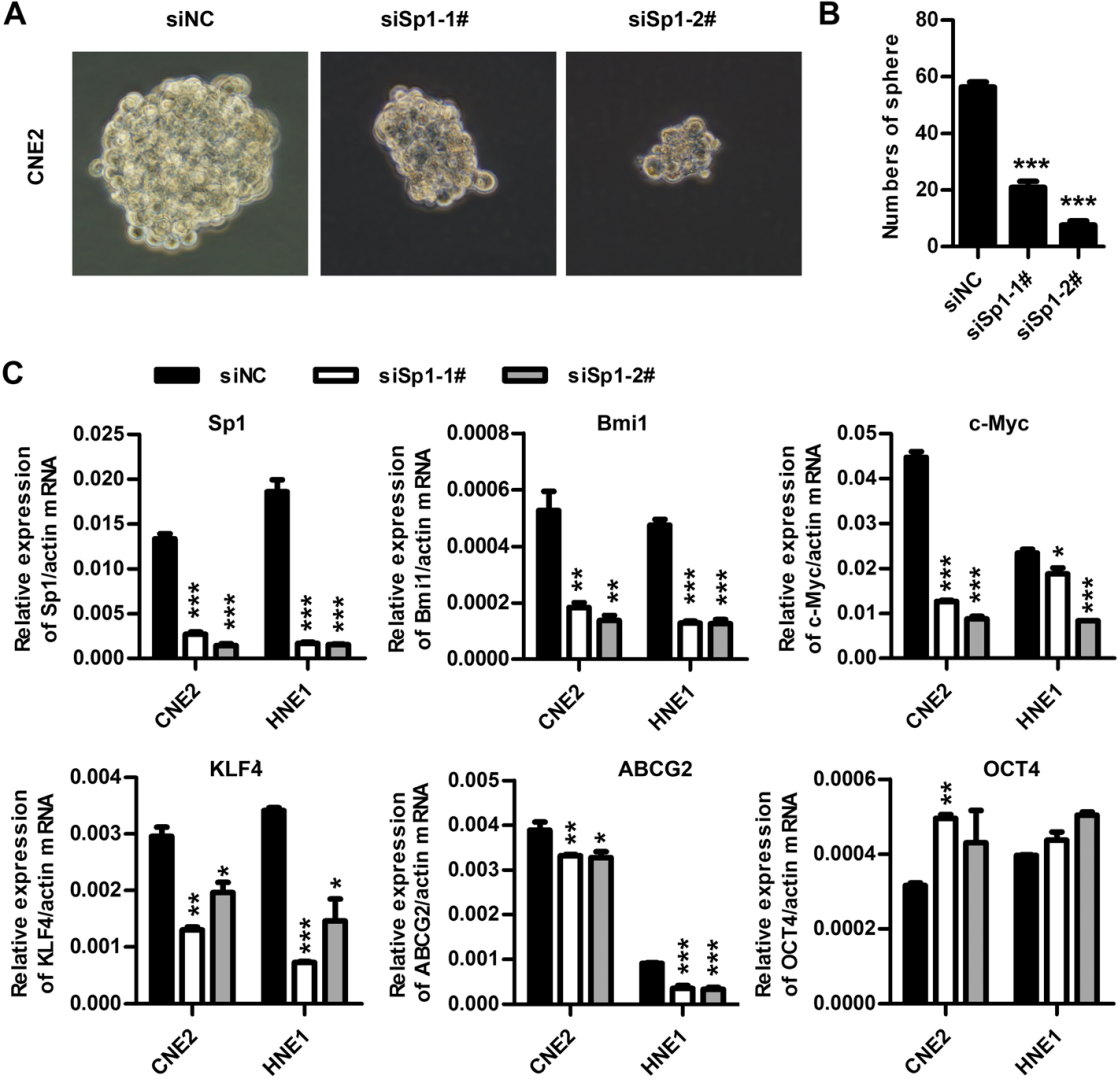
**Knockdown of Sp1 suppressed the sphere formation and the expressions of stem cell markers of NPC cells. (A and B)** Knockdown of Sp1 repressed the sphere formation of CNE2 cells. CNE2 cells transfected with either siSp1-1#, siSp1-2# or siNC were grown in tumor sphere medium for 7 days. Colonies were photographed at 200× magnification and were counted at 40× magnification. *** *P* < 0.001. **(C)** The effect of Sp1 knockdown on the expressions of stem cell transcription factors. CNE2 and HNE1 cells transfected with siSp1-1#, siSp1-2# or siNC for 72 h were analyzed for the levels of stem cell transcription factors by qRT-PCR. The expression level of the indicated gene was normalized to β-actin. * *P* < 0.05, ** *P* < 0.01, *** *P* < 0.001.

Stem cells can be expanded as sphere-like cellular aggregates in the sphere medium containing EGF and bFGF. To further determine the role of Sp1 silencing in the stem-cell like phenotype of NPC cells, sphere formation potential was monitored. CNE2 cells transfected with the indicated siRNA duplexes were cultivated in the sphere medium for 7 days. The sphere formation number was significantly lower in Sp1 silenced CNE2 cells (Figure 6A and B), suggesting knockdown of Sp1 may suppress the stem-cell like phenotype in CNE2 cells. Bmi1, c-Myc, KLF4, SOX2, NANOG and OCT4 are the key stem cell transcription factors (SCTFs). ABCG2, an ABC transporter member, is associated with the formation of side population and stem/progenitor features in nasopharyngeal carcinoma [38]. We therefore determined whether knockdown of Sp1-suppressed the stem-cell like phenotype was associated with changes in these molecules. Bmi1, c-Myc, KLF4 and ABCG2 were all decreased in Sp1-knockdown CNE2, HNE1 and HK1 cells (Figure 6C). In contrast, OCT4 and SOX2 was unaffected by Sp1 knockdown, indicating down-regulation of Sp1 suppressed the stem-cell like phenotype, depending on the reduced expression of Bmi1, c-Myc, KLF4, and ABCG2, but not OCT4 and SOX2 (data not shown).

Taken together, our data suggest that Sp1 was enriched in NPC cell lines as well as tissues and are highly correlated with the NPC progression. Down-regulation of Sp1 may induce the expression of p27 and p21, as well as the expressions of the critical SCTFs, resulting in the suppression of cell proliferation, clonogenicity, anchorage-independent growth and the stem-cell like phenotype in NPC cells. These findings demonstrated the potential use of Sp1 inhibitor in the clinical therapy of nasopharyngeal carcinoma.

## Discussion

Although Sp1 has been investigated extensively in multiple types of cancers, the level of Sp1 in nasopharyngeal carcinoma and molecular mechanisms by which Sp1 modulates the behavior of tumor cells remain elusive. In the present study, we revealed that higher level of Sp1 correlates with advanced tumor stage in nasopharyngeal carcinoma*.* Down-regulation of Sp1 suppressed cell growth, the G1/S phase transition, clonogenicity and anchorage-independent growth of NPC cells. Sp1 exert a specific role on the expression of genes related to cell proliferation and clonogenicity, such as p27, p21, Bmi1, c-Myc, KLF4 and ABCG2. Taken together, these results suggest a fundamental role of Sp1 in the phenotypic regulation of cancer cells, and implicate the potential application of Sp1 in cancer therapy.

Sp1 has been extensively investigated in multiple cancers [26]. However, the significance of Sp1 in human head and neck cancers, such as nasopharyngeal carcinoma, has never been explored. In the present study, the pivotal roles of Sp1 in the cell proliferation, clonogenicity and anchorage-independent growth were confirmed in CNE2 and HNE1 or HK1 cells. G1/S phase transition is regulated by a balance of cyclins and cyclin-dependent kinase inhibitors. Cyclins (e.g., cyclin D1) facilitate S-phase entry, whereas cyclin-dependent kinase inhibitors (e.g., p21 and p27) keep cells arrested in G1 phase. We found knockdown of Sp1 significantly promoted the expressions of p21 and p27 in both CNE2 and HNE1 cells, but had no obvious effect on the expressions of CDK4, suggesting suppression of Sp1 promoted cell arrest in G1 phase though the elevated levels of p27 and p21. Furthermore, down-regulation of Sp1 may suppress the acquisition of cancer stem cell phenotypes through the reduced expressions of SCTFs, including Bmi1, c-Myc and KLF4. Taken together, Sp1 promotes proliferation, clonogenicity and anchorage-independent growth of NPC cells.

In addition to being as an oncogene, Sp1 can also act as a tumor suppressor in various types of cancer. Chuang et al. reported that Sp1 overexpression suppressed the cell growth and increased the sub-G1 fraction, caspase-3 cleavage, and annexin-V signal in HeLa and A549 cells. When cells entered the mitotic stage, Sp1 overexpression could induce p53-dependent apoptosis through affecting mitotic chromatin packaging. Moreover, Hsu reported that the proportion of low Sp1 expression in patients with stage IV lung adenocarcinoma was higher than that in patients with stages I and II of lung adenocarcinoma. Sp1 negatively correlated with poor prognosis. Sp1 level accumulated strongly in early stage and was required for lung tumor growth, but it was declined in late stage and suppressed metastasis through inducing E-cadherin expression. Therefore, the role of Sp1 in tumor development is paradox and variable, largely depending on the cellular context.

We previously reported that Sp1 activates the transcription of Bmi1 and CENPH in nasopharyngeal carcinoma [32],[33]. Both Bmi1 and CENPH are oncogenes which are elevated in various malignancies originating in the breast, esophagus and nasopharynx [39]–[41]. Higher levels of Bmi1 and CENPH are correlated with an advanced stage and/or unfavorable prognosis. Bmi1, a member of the polycomb group, promotes tumor progression by inhibiting the transcription of tumor suppressors, such as p53 [42], p21 [43], INK4a and p19^Arf^[44]. CENPH, a basic component of the constitutive centromere-associated network, induces continuous chromosome instability during mitosis, which is found in the earliest stages of tumorigenesis [39]. Therefore, the cancer-promoting role of Sp1 may also be mediated by transcriptional activation of its downstream genes, such as Bmi1 and CENPH.

MITA, an aureolic acid-type polyketide isolated from streptomyces, specifically inhibits binding of Sp1 to GC-rich DNA and thus suppressed the Sp1-targeted genes mediating proliferation, angiogenesis, invasion and metastasis [45]. It has been used in the treatment of various malignancies, including testicular carcinoma [46], osteolytic myelomatosis [47], pancreatic cancer [48]. However, the role of MITA in NPC has never been explored. In this study, MITA was found to significantly repress the cell viability of both CNE2 and HNE1 cells, indicating Sp1 may be the potential target in the clinical therapy of nasopharyngeal carcinoma.

In summary, we investigated the expression level and potential role of Sp1 in nasopharyngeal carcinoma and its underlying mechanisms. Our data revealed that higher level of Sp1 may play important role in the development of nasopharyngeal carcinoma and highlighted the potential use of Sp1 inhibitor in the clinical therapy of NPC.

## Conclusions

In the present study, we found the level of Sp1 was elevated in cancerous than in normal primary NPECs, and was higher in NPC tissues with advanced clinical stage than that with early clinical stage. Either inhibition of Sp1 activity by mithramycin A, the FDA-approved chemotherapeutic anticancer drug or knockdown of Sp1 by siRNA duplex suppressed the growth of NPC cells. Furthermore, we demonstrated that silencing of Sp1 inhibited cell proliferation, clonogenicity, anchorage-independent growth and the stem-cell like phenotype of NPC cells through inducing the expression of p27 and p21, and through impairing the expressions of SCTFs, including Bmi1, c-Myc, KLF4, NANOG and OCT4 in NPC cells. These findings highlight the significance of Sp1 down-regulation in suppressing cell proliferation, clonogenicity, anchorage-independent growth and the stem-cell like phenotype, implicating Sp1 as an attractive candidate for NPC therapy.

## Abbreviations

Bmi1: B-lymphoma mouse Moloney leukemia virus insertion region 1

CENPH: Centromere protein H

IHC: Immunohistochemistry staining

MITA: Mithramycin A

NPC: Nasopharyngeal carcinoma

NPEC: Nasopharyngeal epithelial cells

NKUC: Non-keratinizing undifferentiated carcinoma

NKDC: Non-keratinizing differentiated carcinoma

qRT-PCR: Quantitative Real-time RT-PCR

SCTFs: Stem cell transcription factors

## Competing interests

The authors declare no competing interests.

## Authors’ contributions

JPZ participated in the design of the study, performed the statistical analysis, and drafted the manuscript. HZ performed real-time PCR experiments and Western blot. HBW carried out the key experiments, performed statistical analysis, prepared figures and tables, and drafted the manuscript. YXL carried out immunohistochemistry staining. GHL participated in the cell culture. SX performed flow cytometry analysis. MZL provided important comments in the cell culture. MSZ conceived of the study, and participated in its design and coordination and helped to draft the manuscript. All authors read and approved the final manuscript.
